# Prevalent diabetes and long-term cardiovascular outcomes in adult sepsis survivors: a population-based cohort study

**DOI:** 10.1186/s13054-023-04586-4

**Published:** 2023-07-31

**Authors:** Federico Angriman, Patrick R. Lawler, Baiju R. Shah, Claudio M. Martin, Damon C. Scales, the Sepsis Canada Network

**Affiliations:** 1 Department of Critical Care Medicine, Sunnybrook Health Sciences Centre, Toronto, ON Canada; 2 Interdepartmental Division of Critical Care Medicine, University of Toronto, Toronto, ON Canada; 3 McGill University Health Centre, Montreal, Canada; 4 Peter Munk Cardiac Centre, University Health Network, Toronto, Canada; 5 ICES, Toronto, Canada; 6 Institute of Health Policy, Management and Evaluation, Dalla Lana School of Public Health, University of Toronto, Toronto, Canada; 7 Department of Medicine, Sunnybrook Health Sciences Centre, Toronto, Canada; 8 Department of Medicine, University of Toronto, Toronto, Canada; 9 Division of Critical Care, Department of Medicine, Schulich School of Medicine and Dentistry, Western University, London, Canada; 10 Lawson Health Research Institute, London, Canada

**Keywords:** Diabetes, Sepsis survivors, Cardiovascular disease, Myocardial infarction, Stroke, Competing risks

## Abstract

**Background:**

Sepsis survivors are at elevated risk for cardiovascular disease during long-term follow-up. Whether diabetes influences cardiovascular risk after sepsis survival remains unknown. We sought to describe the association of diabetes with long-term cardiovascular outcomes in adult sepsis survivors.

**Methods:**

Population-based cohort study in the province of Ontario, Canada (2008–2017). Adult survivors of a first sepsis-associated hospitalization, without pre-existing cardiovascular disease, were included. Main exposure was pre-existing diabetes (any type). The primary outcome was the composite of myocardial infarction, stroke, and cardiovascular death. Patients were followed up to 5 years from discharge date until outcome occurrence or end of study period (March 2018). We used propensity score matching (i.e., 1:1 to patients with sepsis but no pre-existing diabetes) to adjust for measured confounding at baseline. Cause-specific Cox proportional hazards models with robust standard errors were used to estimate hazard ratios (HR) alongside 95% confidence intervals (CI). A main secondary analysis evaluated the modification of the association between sepsis and cardiovascular disease by pre-existing diabetes.

**Results:**

78,638 patients with pre-existing diabetes who had a sepsis-associated hospitalization were matched to patients hospitalized for sepsis but without diabetes. Mean age of patients was 71 years, and 55% were female. Median duration from diabetes diagnosis was 9.8 years; mean HbA1c was 7.1%. Adult sepsis survivors with pre-existing diabetes experienced a higher hazard of major cardiovascular disease (HR 1.25; 95% CI 1.22–1.29)—including myocardial infarction (HR 1.40; 95% CI 1.34–1.47) and stroke (HR 1.24; 95% CI 1.18–1.29)—during long-term follow-up compared to sepsis survivors without diabetes. Pre-existing diabetes modified the association between sepsis and cardiovascular disease (risk difference: 2.3%; 95% CI 2.0–2.6 and risk difference: 1.8%; 95% CI 1.6–2.0 for the effect of sepsis—compared to no sepsis—among patients with and without diabetes, respectively).

**Conclusions:**

Sepsis survivors with pre-existing diabetes experience a higher long-term hazard of major cardiovascular events when compared to sepsis survivors without diabetes. Compared to patients without sepsis, the absolute risk increase of cardiovascular events after sepsis is higher in patients with diabetes (i.e., diabetes intensified the higher cardiovascular risk induced by sepsis).

**Supplementary Information:**

The online version contains supplementary material available at 10.1186/s13054-023-04586-4.

## Introduction

Sepsis is conceptualized as a life-threatening acute organ dysfunction in response to infection and represents a leading cause of morbidity and mortality worldwide [1–5]. Acute complications of sepsis may be related to both the infection itself and the host’s response and are characterized by distinct organ failures and high risk of mortality [3, 5, 6]. Moreover, there is an increasing awareness of the long-term health risks of sepsis, including—but not limited to—recurrent sepsis, clinical deconditioning and re-hospitalization, mental health problems, and cardiovascular events (which can be considered as part of the so-called post intensive care syndrome) [7–17].

Social determinants of health and baseline burden of disease such as nutrition, lifestyle choices, and comorbid conditions may all interact with the risk of both short- and long-term outcomes following sepsis [6, 18–23]. Diabetes mellitus may be a particularly important determinant of sepsis-related outcomes due to the associated cardiovascular changes and its high and ever-increasing prevalence [24–26]. Diabetes may affect post-sepsis cardiovascular disease either (1) directly, (2) through its association with other comorbidities such as hypertension, (3) through the varying severity of the sepsis episode, or (4) through the amplification of changes (e.g., inflammation cascade) that may occur after sepsis [23, 27]. However, once sepsis develops in patients with diabetes, the impact on organ failure, in-hospital events, and long-term clinical outcomes remains unclear [28, 29]. Overall, short-term mortality and risk of acute lung injury following sepsis may be reduced compared to patients without diabetes, while acute renal failure appears to be more common [23, 25, 30–33]. Diabetes is an established risk factor for cardiovascular disease, but the degree to which diabetes increases the risk of experiencing cardiovascular outcomes after sepsis (e.g., the potential additive risk in sepsis survivors) remains incompletely characterized [23, 27].

We sought to describe the association of pre-existing diabetes with long-term cardiovascular outcomes in adult sepsis survivors using population-based data from the province of Ontario. We hypothesized that pre-existing diabetes would be associated with a higher risk of cardiovascular disease in sepsis survivors.

## Methods

### Data sources and study population

We created the study cohort using population-based provincial health administrative databases contained at ICES, an independent, non-profit research institute whose legal status under Ontario’s health information privacy law allows it to collect and analyze healthcare and demographic data, without consent, for health system evaluation and improvement. These datasets were linked using unique encoded identifiers. Our study was developed in accordance with the amended Declaration of Helsinki, and this report follows the Strengthening the Reporting of Observational Studies in Epidemiology (STROBE) [34]. The use of data in this project was authorized under Sect. 45 of Ontario’s Personal Health Information Protection Act, which does not require review by a Research Ethics Board.

Our cohort included adults (age 18 years or older) in the province of Ontario, Canada, who survived a first sepsis-related hospitalization between April 2008 and April 2017. The study dates were chosen to optimize data completeness, and to allow a minimum follow-up of one year for all patients (to March 2018). Sepsis was identified using a previously validated algorithm [35, 36]. To control for baseline confounding, patients with pre-existing cardiovascular disease (identified during a 5-year lookback period) were excluded [14]. For all patients, the start of follow-up (i.e., index date) was defined as the date of hospital discharge. Patients were followed until outcome occurrence up to a maximum of five years or end of the study period.

### Main exposure and outcomes of interest

Our exposure of interest was pre-existing diabetes at the time of sepsis hospitalization, defined using a previously validated algorithm [26]. This algorithm has high sensitivity and specificity but does not differentiate between type 1 or type 2 diabetes mellitus [26]. The composite primary outcome of interest was comprised of any of myocardial infarction, stroke, or cardiovascular death, defined using International Classification of Diseases 10-CA codes [37–39]. Secondary outcomes of interest included myocardial infarction, stroke, recurrent sepsis within 1 year, and the competing risk of non-cardiovascular death [14]. Table S1 in the supplement describes specific coding strategies used to define main variables of interest; details can be found elsewhere [14, 26, 35, 39].

### Statistical analysis

Patients’ demographic, clinical, and hospital level characteristics were summarized using proportions for categorical variables and mean and standard deviation (SD) or median and interquartile range (IQR) for continuous variables, as appropriate. Baseline characteristics of patients with or without diabetes were compared using standardized mean differences (SMD) [40]. SMD greater than 10% were considered relevant.

We used propensity score matching to control for measured confounding [40]. Specifically, we created a propensity score (i.e., disease risk score) for pre-existing diabetes using a logistic regression model including the following measured confounders identified using subject matter knowledge: age, sex, income quintile, long-term care residency, classic cardiovascular risk factors such as hypertension, dyslipidemia, and atrial fibrillation, and baseline comorbid conditions such as chronic kidney and pulmonary disease. To avoid overmatching on hospital characteristics that may lie within the causal pathway between pre-existing diabetes and cardiovascular disease, we did not match for severity of sepsis or intensity of organ support [14]. We then performed one to one greedy matching without replacement with a caliper width of 0.15 on the logit scale [40]. Outcome occurrence was summarized using cumulative incidence during long-term follow-up, alongside cumulative incidence functions [41]. To estimate the association between diabetes and time to (first) binary outcomes, we used cause-specific Cox proportional hazards model with robust standard errors based on the sandwich estimator to account for the matching procedure [42]. To estimate the association between diabetes and recurrent sepsis during the first year following hospital discharge (and to allow for multiple or recurring events), we used a Poisson regression model [43], also with robust standard errors. Effect estimates are reported as hazard ratios (HR) or incidence rate ratios (IRR) as appropriate, alongside 95% confidence intervals.

### Secondary and sensitivity analyses

To further explore the impact of diabetes on long-term cardiovascular disease after sepsis, our main secondary analysis explored the modification of the association between sepsis and subsequent cardiovascular events by pre-existing diabetes. Specifically, we explored whether the previously identified effect of sepsis on cardiovascular disease during long-term follow-up varied across subgroups of patients with and without diabetes [14]. For this analysis, and in a similar way to previously reported [14], we included matched adult patients who survived a hospitalization (either related to sepsis or not). Details about cohort creation, matching, and the comparison between patients with and without sepsis can be found in the supplement and elsewhere [14]. We then fitted a multivariable Cox proportional hazards model (i.e., on the multiplicative scale) with sepsis, pre-existing diabetes, and their interaction as main covariates. To further assess effect measure modification on the additive and multiplicative scales, we also fitted generalized linear models with a binomial distribution and an identity or a log link to estimate absolute risk differences (i.e., additive scale) and risk ratios (i.e., multiplicative scale), respectively [44]. The presence of effect measure modification in all models was assessed based on a Wald test for the interaction term, and the effect of sepsis on long-term cardiovascular disease is reported separately for patients with and without pre-existing diabetes. Further details can be found in the supplement.

We also conducted several sensitivity analyses to assess the robustness of our main findings. First, we report the *E*-value for the point estimate and lower bound of the 95% confidence intervals for our main analysis on the primary outcome of interest [45]. Second, we utilized Fine and Gray models to take into account the competing risk of non-cardiovascular death, reporting sub-distribution HRs alongside 95% CI [46]. Third, since patients with chronic kidney disease may have a differential risk of sepsis and subsequent outcomes, we refitted our analysis restricting to patients without kidney failure at baseline (identified using International Classification of Diseases 10-CA codes during the lookback period) [47]. Fourth, we planned to further adjust, if needed, for baseline imbalances after matching (i.e., SMD greater than 10%) [48]. Fifth, since the intensity of the acute illness may modify the long-term cardiovascular risk following sepsis, we re-fitted our analysis while adjusting for in hospital characteristics (e.g., receipt of renal replacement therapy and mechanical ventilation) [23]. Further, since renal replacement therapy has been recently recognized as a potential risk factor of cardiovascular disease in sepsis survivors, we performed a causal mediation analysis considering the receipt of new dialysis as a potential mediator of the association between diabetes and the primary outcome (further details found in the supplement) [23, 49]. Sixth, we evaluated the impact of preadmission glycemic control of patients with diabetes on subsequent cardiovascular disease. To this end, we stratified patients by their measured HbA1c (i.e., less than 6.5%, 6.5% to 7.9%, more or equal than 8%). Seventh, we re-fitted our estimates considering those patients without previously identified diabetes but with a baseline HbA1c greater than or equal than 6.5% as having pre-existing diabetes. Eighth, we conducted a quantitative bias analysis to correct for potential misclassification of the exposure based on the expected accuracy of the coding algorithm for pre-existing diabetes [50]. Finally, we re-fitted our main analysis without the exclusion of patients with pre-existing cardiovascular disease since they may represent a population specifically at risk of subsequent cardiovascular outcomes after sepsis.

All analyses were performed using SAS Enterprise Guide version 7.1 (Cary, NC) and STATA v.14.2 (StataCorp, College Station, TX). A p-value of 0.05 was used as threshold for statistical significance and all tests were two-sided.

## Results

**Fig. 1:**
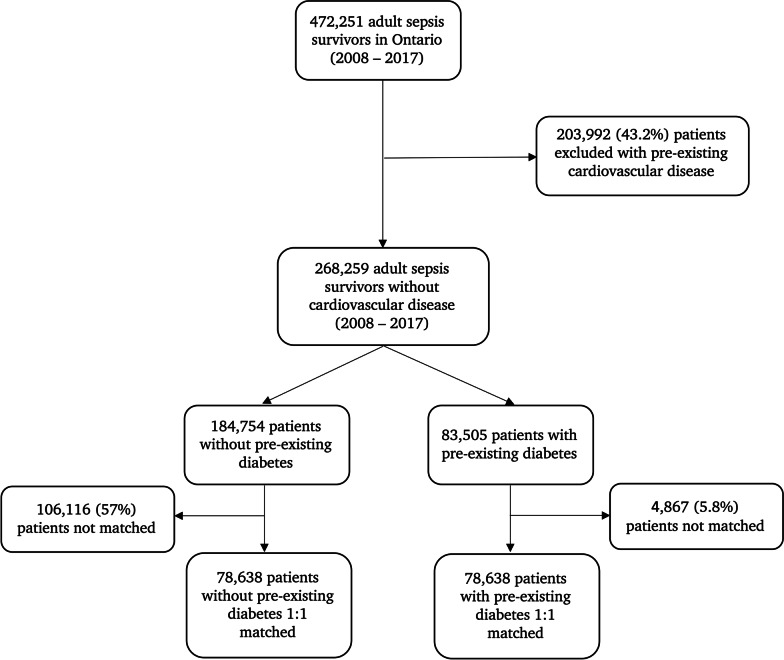
Study flowchart. Cohort creation based on 1:1 matching on a propensity (i.e., disease-risk) score

Overall, 78,638 sepsis survivors with pre-existing diabetes were matched to sepsis survivors without diabetes during the study period (Fig. 1 and Table 1; Additional file 1: Table S2 shows the baseline characteristics of the complete unmatched sample). Mean age of patients was 71 years, and 55% were female. Median duration since diabetes diagnosis was 9.8 years (IQR 3.9–15.3); mean HbA1c on the last blood work prior to admission (available for 76% of sample) was 7.1% (SD: 1.6). Main comorbidities in patients with diabetes were hypertension (79%), active malignancy (28%), dementia (19%), and chronic kidney disease (10%). All demographics and comorbidities were balanced across both groups (i.e., SMD less than 10%).

**Table 1:** Baseline characteristics of matched adult sepsis survivors with or without pre-existing diabetes mellitus in Ontario (2008–2017)

Baseline covariate	Pre-existing diabetes mellitus		SMD
	NO (*N* = 78,638)	YES (*N* = 78,638)	
*Demographics and comorbidities*			
Age (years) – mean, SD	71.3 (14.2)	71.2 (14.4)	0.01
Female sex – %	54.8	54.8	0.00
*Income quintile*^*1*^* – %*			
1	26.3	26.3	0.00
2	22.0	22.0	0.01
3	19.2	19.2	0.01
4	17.5	17.5	0.01
5	14.9	15.0	0.00
Hypertension – %	79.3	79.5	0.00
Dyslipidemia – %	17.5	18.1	0.01
Atrial fibrillation – %	4.3	4.9	0.03
Chronic kidney disease – %	8.9	9.7	0.03
Venous thromboembolic disease – %	2.0	2.3	0.03
Active malignancy – %	27.6	27.8	0.00
Dementia – %	18.6	19.2	0.02
*Sepsis hospitalization characteristics*			
Site of infection			
Pneumonia	31.5	29.9	0.04
Urosepsis	37.1	39.9	0.06
Acute kidney injury – %	11.2	14.9	0.11
Renal replacement therapy – %	1.8	2.3	0.04
Respiratory failure – %	34.3	32.3	0.04
Septic shock – %	23.1	26.5	0.08
Intensive care unit admission – %	18.2	18.7	0.01
Total length of stay (days) – median, IQR	7 (4–16)	7 (4–16)	0.01

^1^Missing for less than 1% of patients

*SMD* Standardized mean difference, *IQR* Interquartile range, *SD* Standard deviation

On average, adult sepsis survivors with and without pre-existing diabetes had similar sources of infection and presence of septic shock during the initial hospitalization (Table 1). No relevant differences were observed in intensive care unit admission or renal replacement therapy (Table 1). Acute kidney injury was more common in patients with pre-existing diabetes (Table 1).

### Major cardiovascular disease and secondary outcomes

**Fig. 2:**
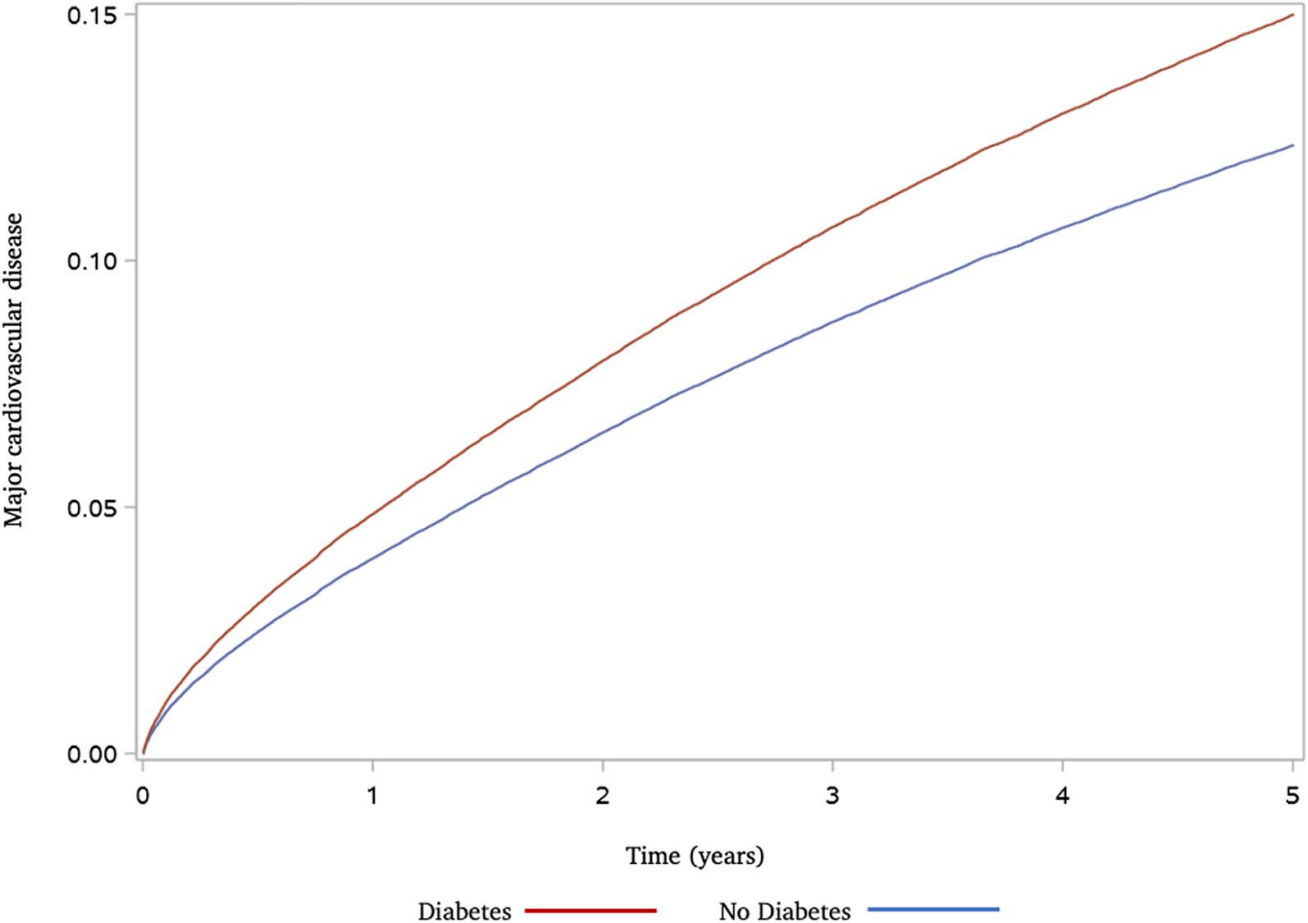
Cumulative incidence function^1^ for the primary composite outcome of myocardial infarction, stroke, or cardiovascular death among adult sepsis survivors in Ontario (2008–2017). Based on a sub-distribution proportional hazards Fine and Gray model including pre-existing diabetes as a binary indicator. Major cardiovascular disease defined as the composite of myocardial infarction, stroke, or cardiovascular death

Participant follow-up information is presented in Additional file 1: Table S3. Median follow-up time for patients with and without pre-existing diabetes was 2.7 years (IQR 1.1–4.9) and 2.8 (IQR 1.2–5.0), respectively. Adult sepsis survivors with pre-existing diabetes experienced a higher hazard of major cardiovascular disease during long-term follow-up when compared to sepsis survivors without diabetes (HR 1.25; 95% CI 1.22–1.29; Table 2). Cumulative incidence functions are shown in Fig. 2. Adult sepsis survivors with pre-existing diabetes experienced a higher hazard of myocardial infarction (HR 1.40; 95% CI 1.34–1.47; Table 2) and stroke (HR: 1.24; 95% CI 1.18–1.29; Table 2). No significant difference was noted for the competing risk of non-cardiovascular death during long-term follow-up (HR 1.02; 95% CI 1.00–1.03; Table 2) or recurrent sepsis during the first year after hospital discharge (IRR 1.01; 95% CI 1.00–1.03; Table 2).

**Table 2:** Main outcome measures in adult sepsis survivors with or without diabetes

Outcome of interest	Hazard ratio^1^ or incidence rate ratio (95% CI)
Myocardial infarction, stroke, or cardiovascular death	1.25 (1.22–1.29)
*Secondary outcomes*	
Myocardial infarction	1.40 (1.34–1.47)
Stroke	1.24 (1.18–1.29)
Competing risk of non-cardiovascular death	1.02 (1.00–1.03)
Recurrent sepsis^2^	1.01 (1.00–1.03)

^1^Based on a cause-specific Cox proportional hazards model including the exposure as a binary variable and using robust standard errors. Shown for all time to binary events (i.e., all except recurrent sepsis)

^2^Recurrent episodes during the first year after hospital discharge. Incidence rate ratio shown, based on a Poisson model with robust standard errors

*CI* Confidence interval

### Modification of the effect of sepsis on cardiovascular disease by diabetes

The effect measure modification analysis is summarized in Table 3, Additional file 1: Table S4 and Table S5. Cohort description, and overall association of sepsis (compared to no sepsis) on cardiovascular disease can be found elsewhere [14]. On the multiplicative scale (i.e., HR or risk ratios (RR)), pre-existing diabetes did not modify the previously estimated effect of sepsis on major cardiovascular disease during long-term follow-up (HR 1.27; 95% CI 1.23–1.31 and HR: 1.31; 95% CI 1.28–1.34 for the effect of sepsis among patients with and without diabetes, respectively; p value for interaction = 0.10; Table 3). Similar results were estimated when using a log-binomial model (RR 1.23; 95% CI 1.19–1.27 and RR: 1.25; 95% CI 1.22–1.28 for the effect of sepsis among patients with and without diabetes, respectively; *p* value for interaction = 0.42; Table 3).

**Table 3:** Modification of the effect of sepsis on major cardiovascular disease during long-term follow-up by pre-existing diabetes^1^

	Cumulative incidence of major cardiovascular events *Sepsis diagnosis*		Association of sepsis (vs. no sepsis) with major cardiovascular events		
	YES	NO	HR (95% CI)^2^	RR (95% CI)^3^	ARD (95% CI)^4^
Patients with diabetes	12.2% (12.0–12.4)	9.9% (9.7–10.1)	1.27 (1.23–1.31)	1.23 (1.19–1.27)	2.3% (2.0–2.6)
Patients without diabetes	9.0% (8.9–9.2)	7.2% (7.1–7.4)	1.31 (1.28–1.34)	1.25 (1.22–1.28)	1.8% (1.6–2.0)

^1^Based on a matched cohort study (1:1) of adult sepsis survivors to survivors of non-sepsis hospitalization, similar to previously reported in Angriman, et al.[14] With similar methodology, exact (on age, sex, and pre-existing diabetes) and propensity score matching was performed. Regression analysis was based on a sample of 249,051 matched pairs. Standard errors based on the sandwich estimator to account for the matching procedure. P values for interaction shown below are based on a Wald test. Major cardiovascular disease defined as the composite of myocardial infarction, stroke, and cardiovascular death. Follow-up from date of hospital discharge up to 5 years

^2^Hazard ratios estimated using a Cox proportional hazards model; *p* value for interaction = 0.10

^3^Risk ratios estimated using a log-binomial model; *p* value for interaction = 0.42

^4^Absolute risk differences estimated using a generalized linear model with an identity link and a binomial distribution; *p* value for interaction < 0.01

*HR* Hazard ratio, *RR* Risk ratio, *ARD* Absolute risk difference, *CI* Confidence interval

The absolute risk (up to 5 years) of cardiovascular disease ranged from 7.2% in patients without sepsis and no pre-existing diabetes to 12.2% among patients who survived a sepsis episode and had prevalent diabetes (Table 3). On the additive scale, pre-existing diabetes modified the effect of sepsis on major cardiovascular disease during long-term follow-up (risk difference: 2.3%; 95% CI 2.0–2.6 and risk difference: 1.8; 95% CI 1.6–2.0 for the effect of sepsis among patients with and without diabetes, respectively; *p* value for interaction < 0.01; Table 3).

### Sensitivity analyses

The main effect estimates were similar across several sensitivity analyses, namely while (1) using Fine and Gray models (sub-distribution HR 1.24; 95% CI 1.21–1.28; Additional file 1: Table S6), (2) restricting to patients without chronic kidney disease (HR 1.21; 95% CI 1.18–1.25; Additional file 1: Table S7), (3) adjusting for acute kidney injury upon admission (HR 1.25; 95% CI 1.21–1.28; Additional file 1: Table S7), (4) adjusting for renal replacement therapy and mechanical ventilation during the index hospitalization (HR 1.25; 95% CI 1.21–1.29; Additional file 1: Table S7), (5) keeping within the analytical sample patients with pre-existing cardiovascular disease (HR 1.21; 95% CI 1.19–1.23; Additional file 1: Table S7), (6) considering those patients without diabetes but with an HbA1c greater than or equal to 6.5% as being exposed (HR 1.24; 95% CI 1.20–1.27; Additional file 1: Table S7), (7) adjusting for potential misclassification of the exposure (RR 1.25; 95% CI 1.20–1.30; Figure S1). Increasing HbA1c at baseline was associated with an increased risk of cardiovascular disease during long-term follow-up (Additional file 1: Table S8). The E-value for the point estimate and lower bound of the main analysis was 1.61 and 1.56, respectively (Figure S2). There was no evidence of relevant mediation of the effect of pre-existing diabetes on subsequent cardiovascular events through the receipt of renal replacement therapy during the sepsis hospitalization (Additional file 1: Table S7).

## Discussion

Our study shows that adult sepsis survivors with prevalent diabetes experience a higher hazard of long-term cardiovascular outcomes compared to similar patients without diabetes. We also observed that sepsis survivors with and without diabetes are at a similarly elevated relative risk for cardiovascular disease compared to patients without sepsis. The absolute risk increase of cardiovascular events after sepsis is higher in patients with diabetes (i.e., on the additive scale, diabetes intensified the higher cardiovascular risk induced by sepsis).

Pre-existing diabetes represents a classic cardiovascular risk factor in the general population, whereas mounting evidence from observational studies show the increased risk of cardiovascular disease in sepsis survivors [14–16, 27, 51–53]. Furthermore, the potential role for diabetes as an independent risk factor for cardiovascular disease in adult sepsis survivors has also been recently highlighted [21, 23, 54]. Prior studies have shown how, among other baseline comorbidities and characteristics of the sepsis episode, pre-existing diabetes may be associated with a higher risk of cardiovascular disease following sepsis; however, these studies were not designed specifically to quantify the impact of diabetes and were undertaken mostly within a prediction framework [21, 23].

A strength of our study is the matching of patients with and without diabetes and exploring several cardiovascular outcomes of interest, while also considering characteristics of the sepsis hospitalization. Our study highlights how diabetes appears to increase the risk of experiencing future cardiovascular outcomes after sepsis but does not increase the risk of other outcomes including recurrent sepsis or all-cause mortality. Furthermore, the presence of additive effect measure modification by diabetes of the association between sepsis and cardiovascular outcomes suggests that, in addition to being considered as independent risk factors, the combination of both carries potentially the highest risk during long-term follow-up. This is in alignment with previous findings showing a differential effect of sepsis on cardiovascular disease introduced by both age and sex [14]. Such groups of sepsis survivors at highest risk of subsequent cardiovascular outcomes could be the focus of future research evaluating potential mitigation strategies [23, 55].

Our study has several limitations. First, sepsis was identified using an administrative algorithm, and some degree of misclassification is expected [36]. This misclassification is more likely to lead to missed sepsis cases but could also lead to some patients being falsely classified as having sepsis with resulting bias toward the null of no association. Similarly, pre-existing cardiovascular disease was defined using administrative coding and some degree of false negative results are expected. Additionally, algorithms do not differentiate between type 1 or type 2 diabetes, and we could not assess differences (if any) between them. Second, our results are subject to residual and unmeasured confounding, where the association between diabetes and cardiovascular disease may be explained by a third characteristic such as baseline frailty (e.g., both associated with diabetes and cardiovascular events) [56]. However, our E-value showed that such a potential unmeasured confounder would need to have a moderate strength of association with both the exposure and outcome to explain our findings [45]. Third, our assessment of long-term outcomes is likely subject to the competing risk of non-cardiovascular mortality, especially in the face of its high occurrence among sepsis survivors [41, 46, 57]; we used formal methods to take this into account and our estimates were robust across different modeling techniques, including Fine and Gray sub-distribution hazards models [46]. Fourth, we did not include information on medications for diabetes (not available in ICES for those patients younger than 65 years of age) that may further modify the cardiovascular risk post-sepsis, either through metabolic control or other pathways yet to be elucidated. This may be of particular importance for the case of metformin given some observational data pointing toward improved outcomes in the critically ill population [58, 59]. In addition, we did not have information on newly diagnosed (e.g., post-sepsis) diabetes after hospitalization that may also influence the risk of long-term cardiovascular disease in sepsis survivors. Whether diabetes is a potential component of the post-intensive care syndrome (and the impact of this on subsequent cardiovascular events in sepsis survivors) remains unknown [60]. Fifth, our outcome assessment was also based on administrative algorithms and as such, some degree of misclassification is expected; however, this is likely non-differential and toward the null of no association [39]. Sixth, we did not capture information on pre-existing obesity, which could affect or modify the risk of cardiovascular disease in adult sepsis survivors with and without diabetes.

In conclusion, sepsis survivors with pre-existing diabetes face a higher long-term hazard of experiencing major cardiovascular outcomes when compared to sepsis survivors without diabetes. Pre-existing diabetes further intensifies the effect of sepsis on major cardiovascular events during long-term follow-up. Future studies should evaluate which subgroups (if any) of patients with diabetes remain at highest risk, and whether improvements in metabolic control and specific prescription patterns can mitigate the cardiovascular risk after sepsis in patients with diabetes.

## Supplementary Information

**Additional file 1. **Supplementary appendix.

## Data Availability

The dataset from this study is held securely in coded form at ICES. While data sharing agreements prohibit ICES from making the dataset publicly available, access may be granted to those who meet pre-specified criteria for confidential access, available at www.ices.on.ca/DAS. The full dataset creation plan and underlying analytic code are available from the authors upon request, understanding that the computer programs may rely upon coding templates or macros that are unique to ICES and are therefore either inaccessible or may require modification.
