# Supplementary material for: Prevalent diabetes and long-term cardiovascular outcomes in adult sepsis survivors: a population-based cohort study
Source: Crit Care. 2023 Jul 31;27:302. doi: 10.1186/s13054-023-04586-4 (PMC10391991; doi:10.1186/s13054-023-04586-4)
Supplement: Supplementary file 1 — Additional file 1. Supplementary appendix. [file 13054_2023_4586_MOESM1_ESM.pdf]

# Prevalent diabetes and long-term cardiovascular outcomes in adult sepsis survivors: a population-based cohort study

Federico Angriman MD MPH PhD; Patrick R. Lawler MD MPH; Baiju R. Shah MD PhD; Claudio M. Martin MD MSc; Damon C. Scales MD PhD
*on behalf of the Sepsis Canada Network*

## Supplementary appendix

**Table S1.** Usual definitions for main variables of interest
**Table S2.** Baseline characteristics of unmatched sample
**Table S3.** Participant follow-up information
**Table S4.** Effect measure modification
**Table S5.** Absolute risk of major cardiovascular disease in different strata
**Table S6.** Fine and Gray models for outcomes of interest
**Table S7.** Additional sensitivity analyses
**Table S8.** HbA1c analysis
**Figure S1.** Bias analysis for misclassification of the exposure
**Figure S2.** E-value analysis
**eReferences**

**Table S1.** Usual definitions for main variables of interest

| Variable of interest | Main components of algorithm (ICD-10 codes unless otherwise specified) | Code significance - Examples |
|---|---|---|
| Sepsis diagnosis(1) | A039, A021, A207, A217, A227, A239, A241, A267, A280, A282, A327, A392, A393, A394, A40, A400, A401, A402, A403, A408, A409, A41, A410, A411, A412, A413, A415, A4150, A4151, A4152, A4158, A418, A4180, A4188, A419, A427, B007, B377, P360, P361, P362, P363, P364, P365, P368, P369, P352, P372, P375, A047, B9548, B956, J189, J440, N390 | Enterocolitis, other sepsis, sepsis due to specific microorganisms, pneumonia, urinary tract infection |
| Severe sepsis(1,2) | R57.2 (septic shock) OR<br>Sepsis codes + J96.0, J96.9, J80, R09.2, R57.0, R57.1, R57.2, R57.8, R57.9, I95.1, I95.9, N17.0, N17.1, N17.2, N17.8, N17.9, K72.0, K72.9, K76.3, F05.0, F05.9, G93.1, G93.4, G93.80, D69.5, D69.6, D65 | Septic shock, acute respiratory failure, cardiogenic shock, shock unspecified, acute renal failure, hepatic failure, delirium, encephalopathy, thrombocytopenia |
| Intensive care unit admission(3) | CCI codes (1.GZ.31.CA-ND; 1.GZ.31.CR-ND; 1.GZ.31.GP-ND); SCU codes (10, 20, 25, 30, 35, 40, 45, 50, 60, 70, 80, 90, 95, 98) | Special care unit codes and procedure codes for mechanical ventilation |
| Pre-existing diabetes(4) | Any patient included in the ODD database | Either type 1 or type 2 diabetes mellitus |
| Myocardial infarction(5) | I21, I22 | Acute myocardial infarction, subsequent ST elevation (STEMI) and non-ST elevation (NSTEMI) myocardial infarction |
| Stroke(5) | I60, I61, I63, I64, H34.1 | Cerebrovascular diseases, nontraumatic intracerebral hemorrhage, cerebral infarction, stroke not specified, central retinal artery occlusion |
| Cardiovascular death(5) | I20, I21, I22, I23, I24, I25, I30, I31, I32, I34, I35, I36, I37, I39, I40, I41, I42, I43, I44, I45, I46, I47, I48, I49, I50, I51, I52, I70, I71, I72, I73, I74, I75 | Death due to acute myocardial infarction, acute ischemic heart diseases, chronic ischemic heart disease, diseases of the pericardium, valvular disease, cardiomyopathy, arrythmias, congestive heart failure, atherosclerosis, peripheral vascular disease, arterial embolism |
| Pre-existing cardiovascular disease(5) | Any patient included in the CHF dataset, I21, I22, I60, I61, I63, I64, H34.1, I70, I73, I23, I24, I25, I00, I01, I02, I05, I06, I07, I08, I09, I30-I43, I46, I51, I71 | Coronary heart disease, prior stroke, peripheral vascular disease, congestive heart failure, and other forms of cardiovascular disease |

**Table S2.** Baseline characteristics of unmatched adult sepsis survivors with or without pre-existing diabetes mellitus in Ontario (2008 – 2017)

| **Baseline covariate** | **Pre-existing Diabetes Mellitus** | | **SMD** |
|---|---|---|---|
| | **NO (N = 184,754)** | **YES (N = 83,505)** | |
| *Demographics and comorbidities* | | | |
| Age (years) – mean, SD | 67.3 (18.7) | 71.1 (14.3) | 0.23 |
| Female sex – % | 59.1 | 54.5 | 0.09 |
| Income quintile[1] – % | | | |
| 1 | 23.1 | 26.5 | 0.08 |
| 2 | 21.0 | 21.9 | 0.02 |
| 3 | 19.3 | 19.1 | 0.00 |
| 4 | 18.4 | 17.2 | 0.03 |
| 5 | 17.7 | 14.6 | 0.08 |
| Hypertension – % | 57.3 | 80.5 | 0.52 |
| Dyslipidemia – % | 15.7 | 17.9 | 0.06 |
| Atrial fibrillation – % | 4.4 | 4.9 | 0.02 |
| Chronic kidney disease – % | 5.4 | 14.4 | 0.30 |
| Venous thromboembolic disease – % | 2.4 | 2.4 | 0.00 |
| Active malignancy – % | 28.2 | 27.6 | 0.01 |
| Dementia – % | 16.8 | 19.0 | 0.06 |
| *Sepsis hospitalization characteristics* | | | |
| Site of infection | | | |
| Pneumonia | 33.3 | 29.4 | 0.09 |
| Urosepsis | 34.7 | 40.5 | 0.12 |
| Acute kidney injury – % | 9.3 | 16.5 | 0.22 |
| Renal replacement therapy – % | 1.4 | 2.9 | 0.10 |
| Respiratory failure – % | 35.9 | 31.7 | 0.09 |
| Septic shock – % | 21.0 | 27.5 | 0.15 |
| Intensive care unit admission – % | 17.0 | 18.8 | 0.05 |
| Total length of stay (days) – median, IQR | 7 (4 – 15) | 7 (4 – 16) | 0.05 |

1. Missing in less than 1% of patients.

SMD: standardized mean difference; IQR: interquartile range.; SD: standard deviation.

**Table S3.** Participant follow-up information.

| **Follow-up** | **Diabetes (N = 78,638)** | **No diabetes (N = 78,638)** |
|---|---|---|
| Follow-up (years) – median, IQR | 2.7 (1.1 – 4.9) | 2.8 (1.2 – 5.0) |
| Myocardial infarction, stroke, cardiovascular death – % | 12.8 | 10.5 |
| Myocardial infarction – % | 5.0 | 3.6 |
| Stroke – % | 4.9 | 4.0 |

Outcome occurrence showed as cumulative incidence (maximum of five years of follow-up)
IQR: interquartile range

**Table S4.** Modification of the effect of sepsis on major cardiovascular disease during long-term follow-up by pre-existing diabetes[1]

| | Effect of sepsis (vs. no sepsis) on major cardiovascular disease | P value for interaction |
|---|---|---|
| ***Multiplicative scale*** | | |
| Hazard ratio (95% CI) | | |
| *Patients without diabetes* | 1.31 (1.28 – 1.34) | 0.10 |
| *Patients with diabetes* | 1.27 (1.23 – 1.31) | |
| Risk ratio (95% CI) | | |
| *Patients without diabetes* | 1.25 (1.22 – 1.28) | 0.42 |
| *Patients with diabetes* | 1.23 (1.19 – 1.27) | |
| ***Additive scale*** | | |
| Absolute risk difference (95% CI) | | |
| *Patients without diabetes* | 1.8% (1.6 – 2.0) | < 0.01 |
| *Patients with diabetes* | 2.3% (2.0 – 2.6) | |

1. Based on a matched cohort study (1:1) of adult sepsis survivors to survivors of non-sepsis hospitalization, as previously reported in Angriman, et al (6). With similar methodology, exact (on age, sex, and pre-existing diabetes) and propensity score matching (on a vector of potential confounders including baseline comorbidities) was performed. Regression analysis based on a sample of 249,051 matched pairs. Hazard ratios estimated using a Cox proportional hazards model; risk ratios estimated using a log-binomial model; absolute risk differences estimated using a generalized linear model with an identity link and a binomial distribution. Standard errors based on the sandwich estimator to account for the matching procedure. P value for interaction based on a Wald test. Major cardiovascular disease defined as myocardial infarction, stroke, and cardiovascular death. Follow-up from date of hospital discharge up to 5 years.
   CI: confidence interval.

**Table S5.** Absolute risk of major cardiovascular[1] disease at 5 years across different strata (considering diabetes and sepsis)

| | | Sepsis diagnosis | |
|---|---|---|---|
| | | YES | NO |
| **Diabetes diagnosis** | YES | 12.2% (12.0 – 12.4) | 9.9% (9.7 – 10.1) |
| | NO | 9.0% (8.9 – 9.2) | 7.2% (7.1 – 7.4) |

1. Based on a generalized linear model with an identity link, a binomial distribution, and robust standard errors. The model included sepsis, baseline diabetes, and their interaction. Cumulative incidence at 5 years and 95% confidence intervals shown for each stratum. Major cardiovascular disease defined as myocardial infarction, stroke, and cardiovascular death.

**Table S6.** Fine and Gray models for main outcomes of interest

| Outcome of interest | Sub-distribution hazard ratio (95% CI)[1] |
|---|---|
| Myocardial infarction, stroke, or cardiovascular death | 1.24 (1.21 – 1.28) |
| *Secondary outcomes* | |
| Myocardial infarction | 1.39 (1.32 – 1.45) |
| Stroke | 1.22 (1.16 – 1.28) |

1. Based on a sub-distribution hazards model on matched cohort.
   CI: confidence interval

**Table S7.** Additional sensitivity analyses

| Analytical approach | Hazard ratio or Risk ratio (95% CI) |
|---|---|
| Restricting to patients without chronic kidney disease | 1.21 (1.18 – 1.25) |
| Adjusting for acute kidney injury | 1.25 (1.21 – 1.28) |
| Keeping patients with pre-existing cardiovascular disease in study sample[1] | 1.21 (1.19 – 1.23) |
| Adjusting for in-hospital characteristics[2] | 1.25 (1.21 – 1.29) |
| Causal mediation[3] | |
| Controlled direct effect | 1.21 (1.18 – 1.24) |
| Adjusting for misclassification of diabetes[4] | 1.24 (1.20 – 1.27) |

1. Based on 314,076 patients (1:1 matched). Patients with pre-existing cardiovascular disease kept in analytical sample.
2. Adjusting for intensity of hospital stay as measured by receipt of renal replacement, mechanical ventilation, and length of stay.
3. Causal mediation using receipt of renal replacement therapy as a potential mediator of the effect of diabetes on subsequent cardiovascular disease after sepsis. Based on generalized linear models using a log link and a binomial distribution. Confidence intervals based on non-parametric bootstrapping (with 500 samples). Risk ratios and 95% CI shown. Percentage mediated ~ 0.5% (95% CI: 0.2% – 0.9%).
4. Considering as diabetes any patients without previously identified diabetes by our coding algorithm but with an HbA1c greater than or equal to 6.5%.

CI: Confidence interval.

**Table S8.** Impact of glycemic control as measured by the HbA1c on long-term cardiovascular disease among adult sepsis survivors with diabetes

| **All patients with at least one HbA1c measurement** | **Cumulative incidence of major cardiovascular events[1]** | | | **Adjusted hazard ratio (95% CI)[2]** | |
|---|---|---|---|---|---|
| | HbA1c < 6.5% | HbA1c 6.5% – 7.9% | HbA1c ≥ 8% | HbA1c 6.5% – 7.9% | HbA1c ≥ 8% |
| Patients with diabetes (N=59,999)[3] | 11.5% | 12.6% | 13.9% | 0.99 (0.94 – 1.04) | 1.32 (1.24 – 1.40) |
| Patients without diabetes (N=35,402)[4] | 9.6% | 9.6% | 15.6% | 0.85 (0.72 – 1.01) | 1.79 (0.85 – 3.77) |

1. Major cardiovascular events defined as myocardial infarction, stroke, and cardiovascular death.
2. Based on a Cox proportional hazards model. HbA1c modelled initially flexibly with a linear and quadratic term. Relationship on the log(hazard) scale approximately linear; then exposure modelled following the levels depicted above. Reference level was defined as HbA1c < 6.5%.
3. 25,112 patients with HbA1c < 6.5%; 22,349 patients with HbA1c between 6.5% and 7.9%; 12,538 patients with HbA1c more or equal than 8%.
4. 33,894 patients with HbA1c < 6.5%; 1,463 patients with HbA1c between 6.5% and 7.9%; 45 patients with HbA1c more or equal than 8%.

**Figure S1.** Bias analysis adjusting for potential misclassification of the exposure[1]

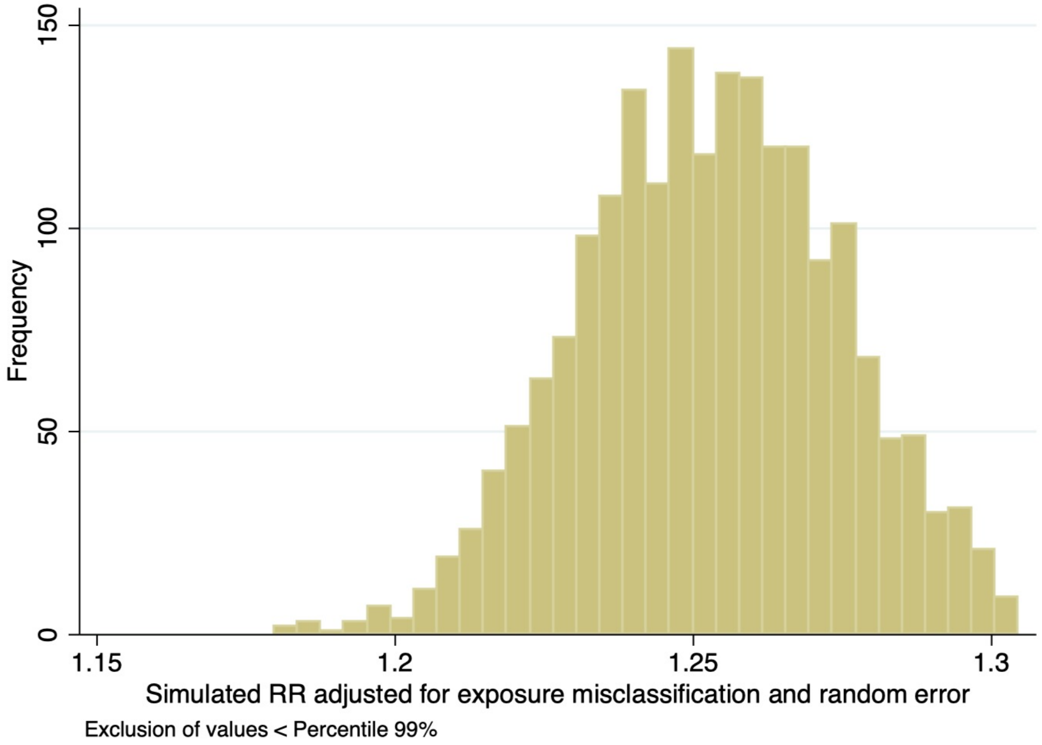


1. Assuming a uniform distribution for sensitivity (0.85 to 0.95) and specificity (0.90 to 0.99) based on the estimated accuracy of the coding algorithm for diabetes. Risk ratio (RR) is for the association between pre-existing diabetes with major cardiovascular events during long-term follow-up. Major cardiovascular events include myocardial infarction, stroke, and cardiovascular death.

**Figure S2.** E-value for the primary composite outcome (i.e., myocardial infarction, stroke, and cardiovascular death)

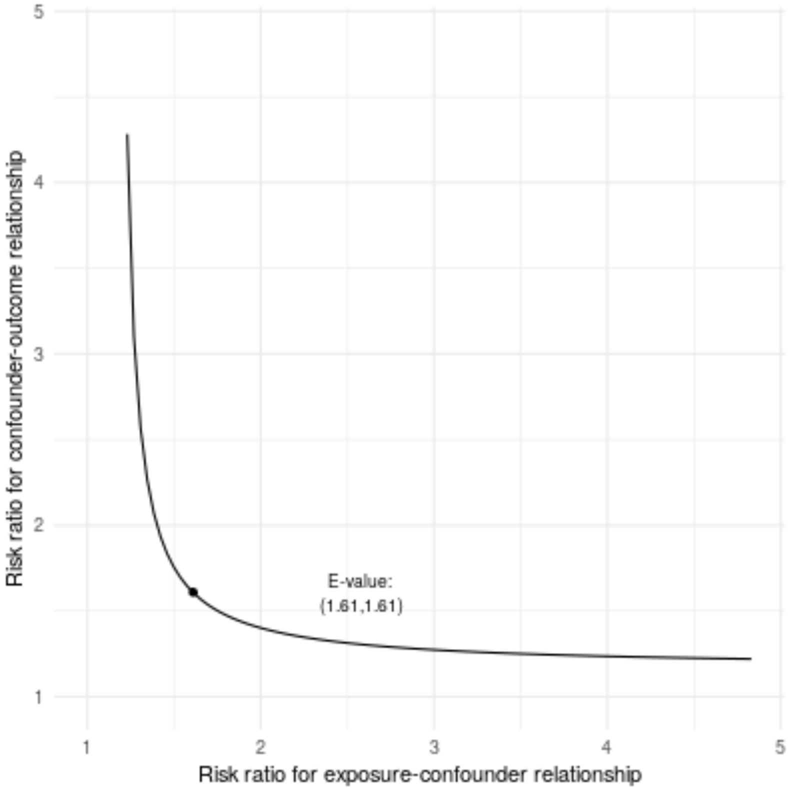


The E-value quantifies the strength of association between an unmeasured confounder and the exposure and outcome that would explain away our findings.

## eReferences


1. Jolley RJ, Sawka KJ, Yergens DW, Quan H, Jetté N, Doig CJ. Validity of administrative data in recording sepsis: A systematic review. Crit Care. 2015;19(1):139.
2. Jolley RJ, Quan H, Jette N, Sawka KJ, Diep L, Goliath J, et al. Validation and optimisation of an ICD-10-coded case definition for sepsis using administrative health data. BMJ Open. 2015;5(12):1–10.
3. Scales DC, Guan J, Martin CM, Redelmeier DA. Administrative data accurately identified intensive care unit admissions in Ontario. J Clin Epidemiol [Internet]. 2006 Aug [cited 2020 Aug 6];59(8):802–7. Available from: https://pubmed.ncbi.nlm.nih.gov/16828673/
4. Hux JE, Ivis F, Flintoft V, Bica A. Diabetes in Ontario Determination of prevalence and incidence using a validated administrative data algorithm. Diabetes Care [Internet]. 2002;25(3):512–6. Available from: http://diabetesjournals.org/care/article-pdf/25/3/512/644465/dc0302000512.pdf
5. Tu J v., Chu A, Donovan LR, Ko DT, Booth GL, Tu K, et al. The Cardiovascular Health in Ambulatory Care Research Team (CANHEART): Using Big Data to Measure and Improve Cardiovascular Health and Healthcare Services. Circ Cardiovasc Qual Outcomes. 2015;8(2):2014–2012.
6. Angriman F, Rosella L, Lawler P, Ko D, Wunsch H, Scales D. Sepsis hospitalization and risk of subsequent cardiovascular events in adults: a population-based matched cohort study. Intensive Care Med. 2022;48(4):448–57.
